# Functional tongue reconstruction with the anterolateral thigh flap

**DOI:** 10.1186/1477-7819-11-303

**Published:** 2013-11-25

**Authors:** Xue Wang, Guangqi Yan, Guirong Zhang, Jiqiang Li, Jihui Liu, Yang Zhang

**Affiliations:** 1 School of Stomatology, China Medical University, No. 117, Nanjing North Street, Heping District, Shenyang, Liaoning 110002, PR China; 2 Stomatology Hospital of Shenyang, No. 138, Zhongshan Road, Heping District, Shenyang, Liaoning 110002, PR China

**Keywords:** Anterolateral thigh flap, Functional outcome, Tongue reconstruction, Microsurgery

## Abstract

**Background:**

A retrospective study was conducted to evaluate the advantages of anterolateral thigh (ALT) flap in tongue reconstruction.

**Method:**

From September 2008 to February 2012, patients receiving ALT flap tongue reconstruction were included in the study. Patients undergoing ALT flap were compared with those undergoing similar surgery with radial forearm flap (RFF). The medical records of the included patients were reviewed, and a questionnaire was used to assess acceptability of the surgery.

**Results:**

All flaps (both ALT and RFF) were successful In the ALT group, most patients were satisfied with the appearance of the reconstructed tongue and the intelligibility of their speech, and there were fewer complications with this technique compared with the RFF.

**Conclusion:**

The ALT flap is an ideal method for tongue reconstruction. The thickness and volume of the ALT flap can be adjusted based on the individual extent of the defect, and it can not only provide bulk but also ensure mobility, and it has other advantages also, including a long pedicle and low donor site morbidity.

## Background

Tongue carcinomas are the most common oral carcinomas [1]. The current treatment strategies for tongue carcinomas are mainly surgery-based comprehensive therapies. There are many methods suitable for defects after ablative surgery or for small or mid-sized defects, including primary closure or local flaps. For large defects, however, reconstruction remains one of the most challenging problems. The tongue plays a key role in speech and deglutition, therefore the ideal reconstructive method should provide not only satisfactory structural cosmesis, but also good restoration of function.

The anterolateral thigh (ALT) free flap was first described by Song *et al*. in 1984 [2]. Wei *et a*l. [3] reported that the failure rate of the ALT free flap was less than 2%, and they concluded that the ALT flap could replace most other flaps for soft tissue, because of the availability of a long pedicle with a suitable vessel diameter, versatility in design, and low donor site morbidity. The ALT flap has since become a standard technique for reconstructive microsurgery, with many reports about its application for reconstruction of the head and neck, upper and lower extremities, and the trunk and breast [4-22], but few reports exist on its use in tongue reconstruction.

Here, we present our experience with the ALT flap for defects of the tongue and floor of the mouth, highlighting the reasons for its versatility and benefits, and presenting analyses of the functional results.

## Methods

### Patients

During the period September 2008 to February 2012, 53 patients underwent simultaneous tumor resection and free ALT flap reconstruction of tongue and mouth floor defects at the Oral-maxillofacial Head and Neck Tumor Center (China Medical University, Shenyang, China). To evaluate the advantages of ALT flap more clearly, we made a comparison between the group receiving ALT and a similar group in which all patients (from September 2008 to February 2012) received radial forearm flap (RFF) reconstruction for defects in the tongue and mouth floor. For each patient, the choice of flap type was based on the surgeon’s experience, defect size, and the patient’s characteristics.

### Surgery

One surgical team resected the tumor, and neck dissection was carried out according to the status of each case. A a plastic surgery team focused on harvesting the tissue for the ALT free flap commensurate with the size of the defect. Perforator detection, dissection, and planning of the ALT flaps were performed as described elsewhere [4,6,7]. First, a line was drawn from the anterior superior iliac spine to the superolateral border of the patella, then the midpoint of this line was determined, because the dominant perforators supplying the flap are located within a circle of 3 cm radius from this point. An incision was made in the sub-fascial plane to identify the location of the perforators and isolate them. At least one of the largest perforators was identified, others can be divided. If necessary, in order tot enhance the reliability of three-dimensional reconstruction, the inclusion of more than one perforator was used, as this improved the safety of the procedure. Once the perforator was confirmed, the skin paddle position could be adjusted appropriately. Depending on the type of ALT free flap needed, the volume and thickness of the could be tailored to the individual extent of the defect.

### Questionnaire

Internal review board authorization was approved for a retrospective chart review. All the medical records were reviewed. At least 6 months after surgery, a functional analysis was conducted. Speech was analyzed postoperatively according to the method described by Song *et al.*[3]. We designed a questionnaire to investigate the differences between the ALT and RFF groups.

### Statistical analysis

SPSS 13.0 was used to analyze the data, and *P* < 0.05 was considered statistically significant.

## Results

In the study, 53 patients underwent reconstruction with the ALT flap. The male–female ratio was 4:1, and the average age was 56.5 years (range: 34 to 70). According to the UICC 2002 TMN staging classification, 10 patients were classified as T2, 27 patients as T3, and the remaining 16t as T4 (Table 1).

**Table 1:** Details of the included patients

**Characteristic**	**ALT flap (n = 53)**	**RFF flap (n = 44)**	***P *** **value**
Age, years; mean (range)	56.5 (34 to 70)	58.0 (32 to 73)	0.875
Male: female ratio, n	42:11	34:10	0.814
Tumor stage			
T2	10	14	0.320
T3	27	20	
T4	16	10	
Flap size, cm^2^	48.5	50.0	0.786
Complications, n	3	2	1.000
Total survival rate, %	100	100	1.000
Post-operative radiotherapy, n	10	8	0.931

*Abbreviations:* ALT, anterolateral thigh; RFF, radial forearm flap.

This patients were compared with 44 patients who underwent reconstruction with RFF. In this group, the average age was 58.0 years. There were 14 patients staged as T2, 20 patients as T3, and 10 patients as T4.

In both groups, there was 100% survival of all flaps. Ten patients in the ALT group and eight patients in the RFF group received post-operative radiotherapy.

During surgery, the type of perforator was identified as the musculocutaneous perforator in 46 patients. In most cases, there was only one perforator used. The flap sizes ranged from 24 cm^2^ (3 × 8 cm) to 84 cm^2^ (7 × 12 cm), with the average flap measuring 48.5 cm^2^ (5.1 × 9.5 cm). Eighteen flaps were harvested as myocutaneous flaps, while the rest were fasciocutaneous flaps. In all flaps, the anatomized vascular vessels were one artery and one vein. There were complications in three flaps within 36 hours after surgeon. A hematoma developed in two flaps; we immediately conducted an exploratory procedure, and found they were caused by non-ligation of venules, which was then corrected. The remaining flap developed vein obstruction; we confirmed that this was due to insufficient anastomosis and re-performed anastomosis again. Despite these problems, all flaps survived.

Speech evaluation showed that 12 patients had good speech intelligibility, 30 patients had acceptable intelligibility, and 11 patients had poor intelligibility.

The results of the questionnaire are shown in Table 2. In general, there were higher levels of acceptability for the ALT flap compared with the RFF, and this was statistically significant for the appearance and level of paresthesia of the donor site.

**Table 2:** Outcomes of questionnaire

**Question**		**Answer**	**Group**		***P *** **value**
			**ALT**	**RFF**	
1	Are you satisfied with the shape of your reconstructed tongue?	Yes	48	40	1.000
		No	5	4	
2	Are you satisfied with the appearance of the donor site?	Yes	53	6	<0.001
		No	0	38	
3	Is there paresthesia in the donor site?	Yes	3	39	<0.001
		No	50	5	
4	Is there weakness in your operated limb?	Yes	2	4	0.406
		No	51	40	

*Abbreviations:* ALT, anterolateral thigh; RFF, radial forearm flap.

## Discussion

Use of the ALT flap is now widespread for head and neck reconstruction. In the majority of cases, the ALT flap is based on the descending branch of the lateral femoral circumflex artery. This artery gives off either musculocutaneous or septocutaneous perforator vessels. A number of authors have reported that the most common perforators were musculocutaneous [4-11], and our study findings (86.8% musculocutaneous) are consistent with this.

In our study, we mainly harvested myocutaneous and fasciocutaneous flaps, because we consider that primary thinning of the ALT flap may increase the risk of failure, as suggested by Sharabi *et al*. [7]. The choice of myocutaneous or fasciocutaneous flap for each patient was based on the size of the defect and the experience of the surgeons. We found that dissection time was somewhat decreased with the ALT myocutaneous flap. We found that harvesting muscle had some important advantages. First, it obliterated dead spaces in the sub-mandible and prevented infections and fistula; in our study, 18 patients with myocutaneous flap had no complications caused by dead space. Second, the volume of the reconstructed tongue is an important factor for swallowing. As the muscular component of the ALT myocutaneous flap could provide adequate bulk, it had a a positive effect in permitting sufficient contact between tongue and palate, thus benefitting the deglutition procedure.

The donor site of the ALT flaps could be repaired by primarily closure, and complications were less frequent than with the RFF (Table 2), suggesting superiority of the ALT flap.

Some modifications of the ALT flap technique are possible. First, the ALT flap can be harvested as a sensation flap through the adjacent anterior branch of the femoral cutaneous nerves. In our previous study [11], we used a sensation ALT flap to reconstruct the defect after parotid malignant tumor in one case, and achieved a satisfactory result. Second, the tensor fascia latae of the ALT flap can enhance its specific properties. In a study conducted by Kuo *et al*. [12], 15 patients with extensive composite defects of the cheek and lip received ALT flaps together with the vascularized fascia and most of them achieved satisfactory outcomes. Third, the ALT flap can be raised as a chimeric flap for three-dimensional reconstruction. In our previous report, we described the reliability of chimeric ALT flaps for soft tissue defects in the head and neck [11].

The main goal of tongue reconstruction is restoration of deglutition and speech. Previous studies [18-20,23] showed that speech intelligibility is closely linked with the mobility of the remaining normal tongue, and that swallowing capacity had a strong relationship with the volume of the reconstructed tongue. Thus, the ideal method for tongue reconstruction should provide both bulk and mobility, and the ALT flap complies with both of these conditions. In our study, most patients achieved satisfactory speech function. However, our experience suggests that this good result can be partially explained by other factors: 1) few patients received post-operative radiotherapy; 2) we focused on achieving a closer resemblance to the three-dimensional shape of the actual tongue [24], and in fact, 90.6% of the cases were satisfied with their reconstructed tongue; 3) we usually harvested an ALT flap that was approximately 20 to 30% larger than the actual defect to allow for tissue atrophy; and 4) we tried to preserve as much of the remaining tongue as possible in every case.

## Conclusions

In summary, we assessed the use of the ALT flap compared with the RFF for defects of the tongue and floor of the mouth. We found that the ALT flap can not only provide bulk but also ensure mobility, along with several other advantages, including availability of a long pedicle and the low donor site morbidity. The thickness and volume of the ALT flap can be adjusted based on the individual extent of the defect. We consider that these benefits make the ALT flap an ideal method for tongue reconstruction. In patients lacking suitable perforators for an ALT flap, an anteromedial thigh flap is a good alternative method [25]. Based on our experience, we recommend that ALT should be the first choice for defects in the tongue and the floor of the mouth.

## Consent

Written informed consent was obtained from the patient for the publication of this report and any accompanying images.

## Competing interests

The authors declare that they have no competing interests.

## Authors’ contributions

XW has made contribution to conception, design, acquisition of information and write this paper; GY, RZ, JL, JL have made contribution to analysis and interpretation of data; YZ have made contribution to help draft the manuscript and revise the paper. All authors read and approved the final manuscript.
